# Trabectedin in metastatic soft tissue sarcomas: Role of pretreatment and age

**DOI:** 10.3892/ijo.2013.1928

**Published:** 2013-05-02

**Authors:** MATHIAS HOICZYK, FLORIAN GRABELLUS, LARS PODLESKA, MARIT AHRENS, BENJAMIN SCHWINDENHAMMER, GEORG TAEGER, CHRISTOPH PÖTTGEN, MARTIN SCHULER, SEBASTIAN BAUER

**Affiliations:** Sarcoma Center, Departments of Medical Oncology, Surgical Oncology and Pathology, West German Cancer Center, University Hospital Essen, University Duisburg-Essen, D-45122 Essen, Germany

**Keywords:** sarcoma, trabectedin, port complication

## Abstract

Trabectedin has mostly been studied in metastatic leiomyosarcoma and liposarcomas. Only limited data are available in other sarcoma subtypes, heavily pretreated and elderly patients. We retrospectively analyzed 101 consecutive sarcoma patients treated with trabectedin at our center. We recorded progression-free survival (PFS), clinical benefit rate (CBR, defined as complete or partial response or stable disease for at least 6 weeks) and toxicity. Covariates were sarcoma subtype, age and pretreatment. On average, trabectedin was administered for 2nd relapse/progression (range 1st to 12th line). A median of 2 cycles and a dose of 1.5 mg/m^2^ (range 1–21 cycles; 1.3–1.5 mg/m^2^) was administered. The median PFS under treatment with trabectedin was 2.1 months in the overall population. Different clinical outcomes were observed with respect to sarcoma subtypes: in patients with L-sarcoma [defined as leiosarcoma and liposarcoma (n=25)] the CBR was 55%. Notably, long lasting remissions were even observed in 7th-line treatment. In contrast, the majority of patients with non-L-sarcomas quickly progressed (median PFS 1.6 months). Nevertheless, a CBR of 34% was achieved, including long-lasting disease stabilization in subtypes such as rhabdomyosarcoma. Patients treated with trabectedin at 1st or 2nd line (n=16) achieved an improved PFS (median 5.7 months, range) and a CBR of 59%. No differences in terms of toxicity or efficacy were observed between patients older than 65 years (n=23) and younger patients (n=78). In this non-trial setting, port-associated complications were more frequent (14%) with trabectedin compared to other continuous infusion protocols administered at our outpatient therapy center. The majority of patients with relapsing L-sarcomas and a substantial fraction of patients with non-L-sarcomas derive a clinically meaningful benefit from trabectedin. Outpatient treatment is well tolerated also in elderly and heavily pretreated patients. Port-associated complications were observed at an unusually high rate. This suggests a drug-specific local toxicity that merits further investigation.

## Introduction

Soft tissue sarcomas represent a heterogeneous group of mesenchymal tumors. While oncogenic mechanisms vary considerably between different histological subtypes soft tissue sarcomas exhibit similar clinical behaviour with regard to metastatic pattern and sensitivity to classical chemotherapy. High grade sarcomas (G2 and G3 based on the FNCLC classification) show moderate response rates between 20 and 30% for single drug and 40–60% for combinational treatments using doxorubicin and ifosfamide (1,2). In 2nd or 3rd line, dacarbacin has been approved based on the data from an early EORTC phase II trial (3).

Trabectedin is a novel chemotherapeutic drug (Ecteinascidin-743, ET743) that was isolated from *Ecteinascidia turbinata*, a tunicate that grows on carribbean mangrove roots. It is a DNA minor groove alkylator that bends DNA towards the major groove (4) and also interferes with several DNA-binding proteins (5). Trabectedin-binding results in inhibition of gene activation, nucleotide excision repair, induction of DNA strand breaks and cell cycle arrest in S and G2 phases (6,7). The transcription of the MDR1 (multidrug resistance) gene, which encodes for the P-glycoprotein, is suppressed by trabectedin, which appears to explain the lack of cross-resistance with other chemotherapeutic drugs (8).

The single agent clinical activity of trabectedin has been shown in pretreated patients with soft tissue sarcomas in several clinical trials (9–12). While trabectedin rarely induces objective remissions (<10%) it shows a substantial progression arrest rate with a favourable toxicity profile which has led to the approval in the European Union for patients with advanced STS after failure of anthracyclines or ifosfamide.

Limited data exist on histological subtypes other than leiomyosarcomas and liposarcomas as well as on patients receiving trabectedin following >2 lines of chemotherapy.

To evaluate the activity and feasibility of trabectedin in a routine clinical setting, including older and heavily pretreated patients, we have conducted a retrospective analysis of 101 consecutive patients at the sarcoma clinic of the West German Sarcoma Center.

## Patients and methods

### Patients

Between 2001 and 2010, 101 patients with histologically proven diagnosis of locally advanced or metastatic soft tissue sarcomas were treated with trabectedin at the Department of Medical Oncology of the West German Cancer Center, University Hospital Essen, Germany.

### Treatment

Patients received an initial dose of 1.5 mg/m^2^ over 24 h as continuous intravenous infusion through a central line (port). Premedication consisted of 20 mg dexamethasone and antiemetic prophylaxis according to our institutional standards. Granulocyte colony-stimulating factor (G-CSF) was not routinely given. Patients had to be fully recovered from any previous treatment-related toxicity (more than grade I CTCAE version 3.0) before administration of the next course. Patients with active viral hepatitis or chronic liver disease or active infection were not treated. Treatment was stopped at clinical or radiological progression, unacceptable toxicity or request by the patient.

### Response evaluation and toxicity

All clinical and laboratory examinations were conducted following our institutional standards and were documented in the patient charts. Imaging was performed after the first 2 cycles and subsequently every 6–9 weeks. The progression-free survival (PFS) time was estimated from start of the first cycle of trabectedin until the documented disease progression. Clinical benefit rate was defined as partial remission or radiologically documented (according RECIST) stable disease for at least 6 weeks. Toxicities were extracted from the patients’ charts and graded according to the National Health Institute (NHI) common toxicity criteria (CTCAE 3.0).

### Statistical analysis

Data are presented as percentage or median plus range unless otherwise specified. Statistical analysis was performed using SPSS 17.0 for Windows (SPSS Inc., Chicago, IL, USA). Survival probabilities were calculated using the Kaplan-Meier method, and differences between the survival curves were assessed using the log-rank test, with p-values <0.05 considered statistically significant.

## Results

The population consisted of 101 patients with a median age at time of first trabectedin of 55 years (range 19–83 years; 55% male/45% female). All patients had confirmed diagnosis of soft tissue sarcoma and included 24 leiomyosarcomas (LMS), 22 liposarcomas (LPS), 13 pleomorphic sarcomas, 14 synovial sarcomas, 6 rhabdomyosarcomas (RMS), and 22 patients with other sarcoma subtypes (Table I).

First-line treatment with trabectedin was given in 5 patients, 2nd-line treatment in 22 patients, 3rd-line treatment in 31 patients and 43 patients received trabectedin later than 3rd-line (Table I).

**Figure 1.**
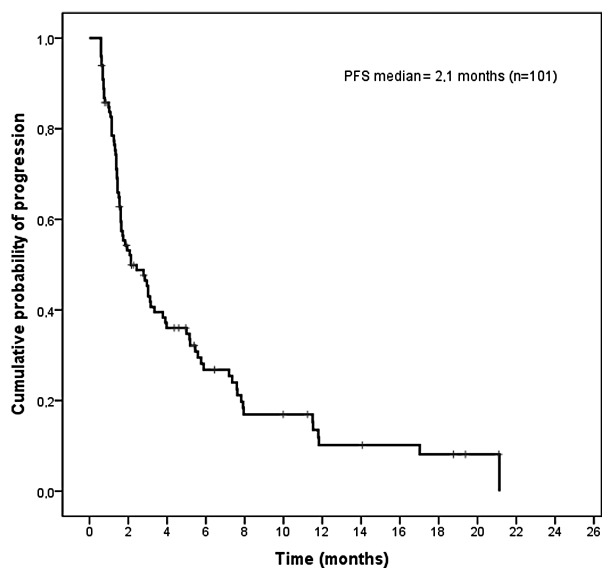
Progression-free survival (months) in all patients calculated from start of trabectedin (n=101).

**Figure 2.**
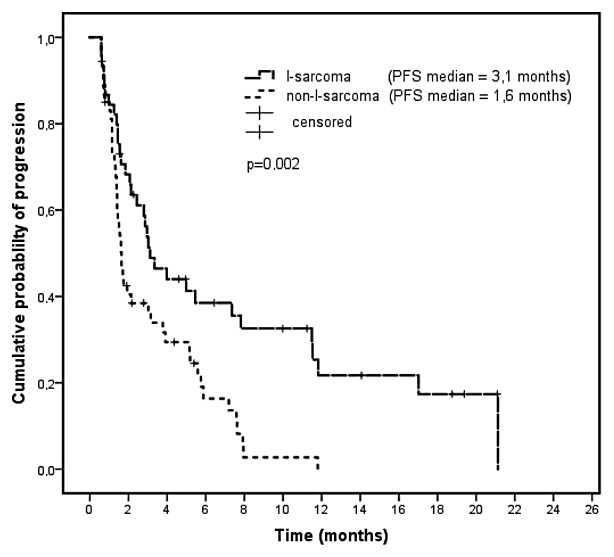
Progression-free survival curves comparing L-sarcomas (n=46) with non-L-sarcomas.

**Figure 3.**
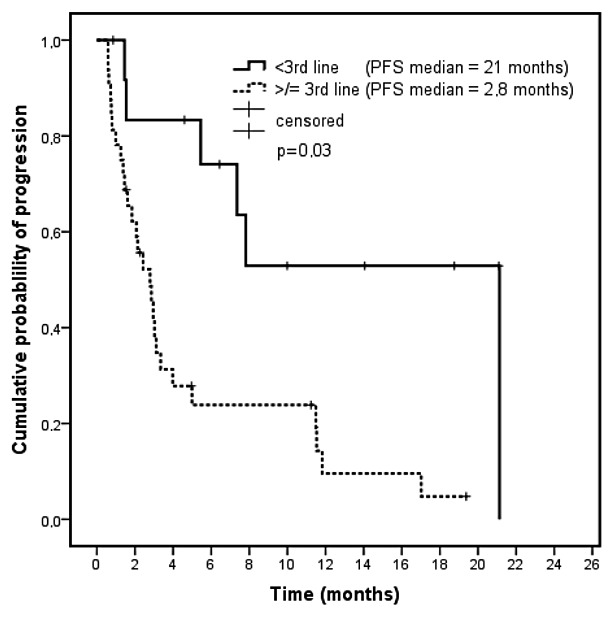
Progression-free survival in L-sarcomas depending on treatment line.

The median PFS of the entire population was 2.1 months (Fig. 1). A median of 2 cycles at a median dose of 1.5 mg/m^2^ (range 1–21 cycles; 1.3–1.5 mg/m^2^) was administered. L-sarcoma patients received trabectedin on average in 3rd-line, with a median PFS of 3 months and a CBR of 55% (Fig. 2). This translated into a 3-month PFS of 51% and a 6 months PFS of 38. Notably, long lasting remissions were even observed in 7th-line treatment. In this subgroup, a significant difference in PFS (21 vs 3 months) was found in patients receiving trabectedin in 1st/2nd-line treatment versus those treated in 3rd or higher lines (p=0.003, t-test) (Fig. 3). Partial remissions were only seen in seven L-sarcomas patients. Of note one leioand one liposarcoma patient, who received trabectedin in the seventh treatment line experienced a disease stabilization of 11 and 19 months (Table II).

**Figure 4.**
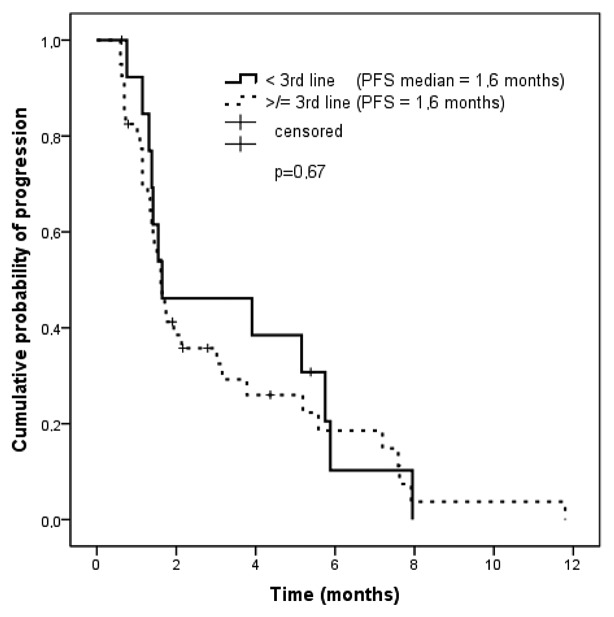
Progression-free survival in non-L-sarcomas depending on treatment line.

In patients with non-L-sarcomas CBR was 35% (n=19) with a median PFS of 1.6 months (Fig. 2). Two of seven RMS patients experienced prolonged stabilization of ≤8 months (pleomorphic, 2) despite heavy pretreatment (Fig. 2). We did not observe a difference in progression-free rates when comparing patients who received trabectedin in earlier (<3rd-line) with those who received it in later treatment lines (Fig. 4).

Dose reductions were necessary in 13 patients, mostly due to hematological toxicities (thrombocytopenia IV°); a dose reduction was performed in one patient receiving trabectedin in 1st line (fatigue grade III), 7 patients in the 2nd line (fatigue grade II, neutropenia grade IV; thrombocytopenia grade III), 3 patients in 3rd line (irritation of port-catheter site; creatinine kinase elevation grade III), 1 patient in the 4th and 6th line each. While toxicities affecting dose intensity did not appear to be more common in pretreated patients, mild hematological toxicities occurred more frequently (Table III).

In the elderly population (>65 years; n=23) trabectedin was given in 3rd line (median) and was well tolerated. Dose reductions were necessary in 3 elderly patients (fatigue CTC grade II and III, leukocytopenia grade IV), no treatment discontinuation was necessary. Seventy-eight percent of all patients received trabectedin in 3rd line or higher (median number of pretreatments, 4 drugs). In these patients, an increased incidence of CTC grade 1–2 hematological toxicities was observed. However, dose reductions were not increased compared to patients receiving trabectedin in 1st- or 2nd-line treatment.

In this non-trial setting, port-associated complications (paravasation, irritation, infection) were more frequent (13%) than in continuous infusion protocols with other drugs (e.g., 5-FU) administered in our large outpatient center.

## Discussion

Patients with metastatic soft tissue sarcomas are still faced with a dismal outcome. For decades only 3 drugs, doxorubicin, ifosfamide, and dacarbacin, have been used, and the median OS has been 12–14 months in these patients (13). Sarcomas occur at all ages and in clinical practice young patients with good performance status often run out of approved chemotherapeutic options. Elderly patients with poor PS have few systemic treatment options that offer a favorable toxicity profile. Trabectedin has recently been approved for treatment of STS after failure of anthracyclins and ifosfamide and has been shown to be particularly active in L-sarcomas.

Here, we present our experience with trabectedin in the setting of a large academic sarcoma center, including a considerable number of elderly, heavily pretreated patients and patients with sarcomas other than LMS and LPS (53%).

With regard to clinical activity, we have seen a median PFS of 3 months and a CBR of 54% in L-sarcomas which compares favorably with published data by Demetri *et al* and Fayette *et al* (14,17).

In our cohort patients with L-sarcomas who received trabectedin in 1st or 2nd line had a better PFS than those who were treated in 3rd or higher line (21 vs 2.7 months). In this context a large clinical trial is currently investigating the role of trabectedin in comparison with doxorubicin in untreated sarcomas (EORTC 62091; Doxorubicin Hydrochloride or Trabectedin in Treating Patients with Previously Untreated Advanced or Metastatic Soft Tissue Sarcoma). One explanation for this difference could be the disproportionate number of patients with myxoid liposarcomas who were treated in the early treatment line (n=4 in 1st or 2nd line). Myxoid liposarcomas appear to be the most sensitive sarcoma subtype (16). Notably long-lasting treatment stabilization was observed even in heavily pretreated patients (7th-line treatment).

In patients with other than lipo- and leiomyosarcoma 58% of the patients exhibited immediate progression after 2–3 cycles of therapy. This might in part be explained by the fact, that these patients had received trabectedin at higher lines than patients with L-sarcomas (non-L-sarcoma >4th line 26%; L-sarcoma >4th line 19%). Also in non-L-sarcomas a considerably number of patients showed prolonged disease stabilization, including subtypes not well known to be trabectedin-sensitive, such as rhabdomyosarcomas (embryonal, 2; pleomorph, 5) or synovial sarcomas. Two patients with rhabdomyosarcoma had a progression-free survival of 7 and 8 months. For non-pleomorphic rhabdomyosarcomas, a phase II trial of trabectedin in pediatric patients has demonstrated a single partial remission but only very limited disease control rate in a pediatric sarcoma population (15).

For non-L-sarcomas we did not see an advantage of treating patients at earlier treatment lines but again observed many patients who exhibited a clinical benefit despite a substantial number of previous lines of chemotherapy.

**Figure 5.**
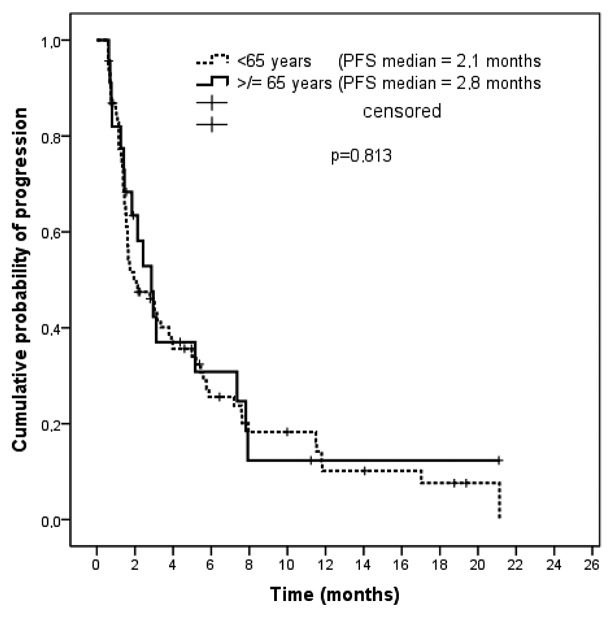
Progression-free survival (in months) in elderly (>65 years) compared to younger patients.

Our cohort included 23 patients >65 years. Interestingly, we did not observe any difference in PFS when compared with patients <65 years (Fig. 5). Also, the use of trabectedin in elderly patients was not associated with decreased dose intensity in our series.

These data underline that trabectedin offers a meaningful alternative in elderly patients in clinical practice. In a similar analysis, Fayette *et al* (17) reported a higher benefit for patients with good performance status (WHO PS 0–1). In our patients the vast majority of patients was treated in an outpatient setting suggesting a good PS in most of them. However, performance status was not a covariate in our analysis. Nonetheless, we have not observed cumulative toxicity and would not preclude treatment from patients with reduced performance status from a treatment with trabectedin.

Despite the limitations of a retrospective analysis the toxicity profile in our patients was comparable to that described in prospective trials (14). Grade III/IV toxicities were a rare exception and only one patient developed a life-threatening complication. In this case, a severe pneumonia requiring mechanical ventilation occurred in a 74-year-old patient following one cycle of trabectedin. Neutropenic fever, as described in the clinical trials, was very rarely seen.

**Figure 6.**
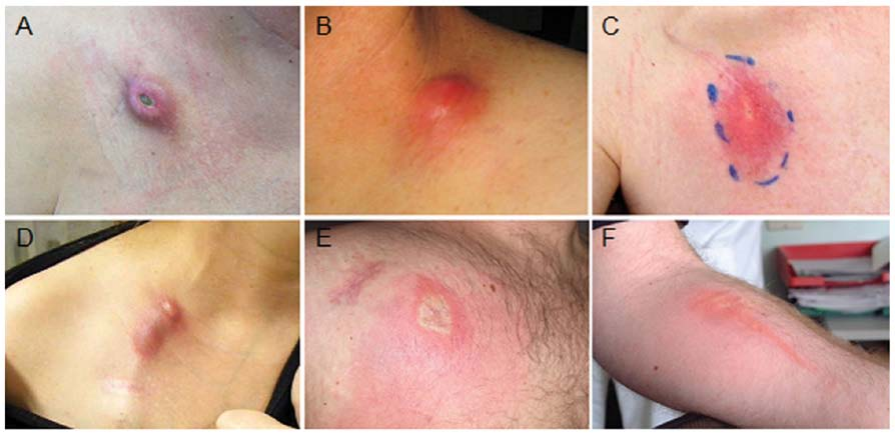
Images of port complication. (A–E) Clinical examples of non-infectious irritation at the port catheter site of the port system. (F) A strong thrombophlebitic reaction after accidental application through a peripheral vein.

A unique side effect that we believe has been underestimated in connection with trabectedin was observed in 14 patients (14%). A non-infectious irritation of the port site with various degrees of severity was a common finding in our clinical practice (Fig. 6). Similar to the case report by Theman *et al* (18) a rash occurred around the port system or along the subcutaneous line leaving the metal port. In many cases, the flow of the port system had been reduced, with small thrombi adjacent to the tip of the port line. Bacterial port infections were unlikely as we usually obtained sterile port cultures and patients did not develop fever. However, the irritation usually caused erythema, pain and itching, which was perceived as very uncomfortable by the patients. The erythema usually persisted for several weeks and then spontaneously faded. In patients with associated port catheter occlusion, usually a new system was implanted. Some patients required several consecutive port replacements, which is extremely rare in patients e.g., with colon cancer who receive 5-FU based treatments in our practice.

While a transvasation of the drug through the port-line cannot be excluded, we hypothesize that an increased resistance of the port system caused by small thrombi at the tip of the port line is associated with a backspill of the drug. Other vesicants, such as doxorubicin or vinorelbin, are usually not given as 24-h infusion outside the hospital environment or oncological practice which might reduce the risk of port-needle translocations.

In contrast to the ‘toxic-looking’ anthracyclins, trabectedin is colorless and paravasations are more difficult to detect both for medical staff as well as for patients. In our experience, a paravasation does not cause immediate irritation or burning sensations. All this may affect the threshold of patients to seek doctor’s advice as well as the reaction of medical staff to aggressively treat paravasations.

We believe that patients should be clearly advised about this side effect and the risk of port dislocation. We have changed our practice in that patients whose port systems show increased resistance or a low spontaneous infusion flow undergo radiological contrast-enhanced imaging to detect port-associated thrombi. If treatment with 10,000 IU of streptokinase does not re-establish a good flow port catheters are replaced.

In conclusion, our analysis confirms that ∼50% of patients with L-sarcomas derive clinical benefit from trabectedin regardless of the extent of pretreatment in a routine setting. Moreover, significant activity is found in a subgroup of patients with non-L-sarcomas including rhabdomyosarcoma. Administration of trabectedin on an outpatient basis is well tolerated in elderly and heavily pretreated patients. The increased incidence of port complications merits further investigation and should be part of patient and medical staff guidance.

## Figures and Tables

**Table I.** Patient characteristics.

Parameter		%
No. of patients	101	100
Median, years (19–83)	53	
<40 years	19	19
40–60 years	50	50
>60 years	32	31
Male	55	55
Female	46	45
Histology	101	100
Leiomyosarcoma	24	24
Liposarcoma	22	22
Pleomorphic sarcoma	13	13
Synovial sarcoma	14	14
Rhabdomyosarcoma	6	5
Other (alveolar sarcoma, chondrosarcoma, desmoplastic small round-cell tumor, epitheloid sarcoma, fibromyxoid sarcoma, MPNST, hemangiopericytoma, no otherwise specified sarcoma)	22	22
Treatment line		
1st line	5	5
2nd line	22	22
3rd or more lines	74	73

Pretreatment included both ifosfamide and doxorubicin; besides gemcitabine and docetaxel were often used; the median age was estimated from date of first trabectedin administration.

**Table II.** Progression-free rates in different sarcoma subgroups.

	L-sarcoma	Non-L-sarcoma	Elderly	Non-elderly
n	46	55	23	78
Median PFS	3.1	1.6	2.9	2.1
3 months PFS	51%	36%	42%	43%
6 months PFS	38%	16%	30%	26%
CBR	55%	34%	43%	44%

CBR, clinical benefit rate (NC+PR).

**Table III.** Adverse events documented according to common toxicity criteria (CTC).

Adverse event	Grade I/II (n)	Grade III/IV (n)
Emesis	40	2
Nausea	75	4
Fatigue	60	5
Fever	20	1
Diarrhea	22	2
Constipation	30	1
Acute renal failure	2	0
Port catheter-associated complications	9	13
Anemia	42	3
Leukocytopenia	13	5
Thrombocytopenia	9	5
AST/AP elevation	36	4

AST, aspartate aminotransferase; AP, alkaline phosphatase.
